# Harnessing Fungi Signaling in Living Composites

**DOI:** 10.1002/gch2.202400104

**Published:** 2024-07-12

**Authors:** Sarah Schyck, Pietro Marchese, Muhamad Amani, Mark Ablonczy, Linde Spoelstra, Mitchell Jones, Yaren Bathaei, Alexander Bismarck, Kunal Masania

**Affiliations:** ^1^ Shaping Matter Lab Faculty of Aerospace Engineering Delft University of Technology Kluyverweg 1 Delft 2629 HS Netherlands; ^2^ Polymer and Composite Engineering Group Institute of Materials Chemistry University of Vienna Waehringer Straße 42 Vienna 1090 Austria

**Keywords:** additive manufacturing, bioelectric signaling, bioinspired materials, engineered living materials, mycelium‐based composites

## Abstract

Signaling pathways in fungi offer a profound avenue for harnessing cellular communication and have garnered considerable interest in biomaterial engineering. Fungi respond to environmental stimuli through intricate signaling networks involving biochemical and electrical pathways, yet deciphering these mechanisms remains a challenge. In this review, an overview of fungal biology and their signaling pathways is provided, which can be activated in response to external stimuli and direct fungal growth and orientation. By examining the hyphal structure and the pathways involved in fungal signaling, the current state of recording fungal electrophysiological signals as well as the landscape of fungal biomaterials is explored. Innovative applications are highlighted, from sustainable materials to biomonitoring systems, and an outlook on the future of harnessing fungi signaling in living composites is provided.

## Graphical abstract

Fungal signaling pathways offer a promising approach in biomaterial engineering, facilitating cellular communication and responses to environmental cues. This review explores fungal biology, signaling networks, and their activation in growth and orientation. Recording fungal electrophysiological signals and the potential of fungal biomaterials are discussed for sustainable applications and biomonitoring systems.

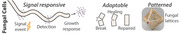

## 1 Introduction

Fungi are remarkable organisms capable of inhabiting virtually any environment due to their unique biochemical processes and ecological adaptabilities. The evolution of species‐specific functions allows them to thrive in environments ranging from dry and sun‐exposed desert soils to deep underwater marine habitats^[^ ^1^ ^]^ as well as develop crucial symbiotic relationships with plants^[^ ^2^, ^3^ ^]^ and animals.^[^ ^4^, ^5^ ^]^ Their widespread colonization of diverse habitats contributed to the evolution of the known fungal biota, which exhibits a wide array of morphologies and biochemical capabilities making them ideal candidates for biotechnological development.^[^ ^6^, ^7^ ^]^ In material science, filamentous fungi attract considerable interest due to their ability to produce large mycelium networks,^[^ ^8^ ^]^ degrade and bind lignocellulosic substrates,^[^ ^9^ ^]^ and develop a continuous polymeric cell wall made of crosslinked β‐glucans and chitin, which has similar mechanical properties to cellulose and other natural polymers.^[^ ^10^ ^]^ These properties mean that biocomposites comprising fungi and lignocellulosic substrates, such as straw, sawdust, and cotton, can be prepared in designer shapes to customizable specifications and geometries.^[^ ^11^ ^]^ However, the material is often heated and pressed to enhance its structural properties, ultimately rendering the fungal component inactive.^[^ ^11^, ^12^ ^]^

A new paradigm in the generation of fungal‐based materials aims to keep the organism alive, leveraging its inherent growth characteristics for manipulation by shaping fungal mycelium into specific forms, engineering by genetically modifying fungi to produce materials with increased strength or resistance to water, and functionalization by using fungal structures to create living sensors or biodegradable electronics. Under living conditions, a fungal biocomposite material exhibits self‐healing and fusing abilities due to the presence of living cells that can grow, multiply, and produce extracellular materials to repair damage or join materials together. While research into living and functional fungal biomaterials remains in its infancy, their potential application for complex processes, such as self‐healing bioconcrete,^[^ ^13^, ^14^, ^15^ ^]^ is of interest.

New research on preparing shapable biomaterials utilizing 3D printing^[^ ^16^ ^]^ represents an important proof of concept. However, the obtained fungal material has limited applications because the printed objects remain elastomeric and can currently serve only nonstructural applications. For example, 3D‐printed hydrogels containing mycelium grow over printed gel to generate shaped living objects that can self‐heal and bridge gaps in the material.^[^ ^16^ ^]^ Mycelium is also capable of growing from printed hydrogels to fill gaps of up to 5 mm, fully fusing separate mycelium blocks.^[^ ^16^ ^]^ The shape freedom of 3D printing facilitates the generation of geometries that fulfil specific engineering requirements, overcoming traditional limitations associated with biomaterial development relying on molds and solid substrates. Damage, such as fractures and holes, heal through stimulated local regrowth of mycelium, achieving 100% restoration of compressive stiffness after the occurrence of damage^[^ ^17^ ^]^ and even self‐healing of leather‐like materials.^[^ ^18^ ^]^

As the boundaries between biological and materials sciences begin to blur, the significance of understanding fungal organization and morphogenesis through their signaling emerges as a prerequisite to the generation of new responsive fungal materials. Recent living microbial materials, such as *Ganoderma* sp. grown on a lignocellulosic substrate, provide an adaptable environment for the growth of modified bacteria *Pantoea agglomerans*, which can be used to propagate biochemical signals.^[^ ^17^ ^]^ In addition to chemical signaling, bioelectric signaling within fungal networks presents an alternative for environmental sensing and regulation. Working within the dearth of literature explaining the origin of fungal signals and how they can be leveraged in functional materials, we explore the biology of fungal networks and their sensing strategies within the context of biosensing applications, in the hope of advancing the development of responsive and adaptive biomaterials.

## 2 Biology of Fungi

Kingdom Fungi comprises eukaryotic organisms with a rich variety of morphologies and life‐cycle strategies, ranging from unicellular budding yeast^[^ ^19^ ^]^ to multicellular filamentous fungi that form mushroom fruiting bodies.^[^ ^20^ ^]^ In their preferred environment, multicellular fungi develop into mature colonies that can generate microscopic reproductive structures, form large spore‐bearing fruiting bodies, or develop large underground mycelium networks connecting the roots of trees.^[^ ^2^, ^20^ ^]^ The array of morphologies achievable in fungi is considerable with known species exceeding 150 000 and estimates of the total number ranging from 2.2 and 3.8 million.^[^ ^6^ ^]^ Fungi, as primary decomposers, are critical to the functioning of most natural habitats. Exploitation of their biological activities has contributed to human development, including the production of food sources, pharmaceuticals, enzymes, metabolites,^[^ ^21^ ^]^ and more recently, engineered living materials.^[^ ^22^ ^]^ As the development of living fungal materials continues, understanding the morphology and physical properties that result from fungal cellular physiological processes will be valuable.

Generally, the structure of macroscopic, multicellular fungi comprises numerous micron‐sized filaments called hyphae which grow, branch out, and interconnect to generate a 3D macroscopic body, called *mycelium*, capable of growing over areas as large as hundreds of kilometers.^[^ ^8^, ^23^ ^]^ Hyphae are filled with cytoplasmic fluid that, coupled with the plasma membrane, maintains hydrostatic pressure within the cell wall and provides turgor to the mycelium.^[^ ^24^ ^]^ The cell wall, which fully covers the hyphal plasma membrane and mainly comprises chitin and glucan, provides an important contribution to the mycelial conformation. Species‐specific genome variability enables the synthesis of additional cell wall components. This leads to the formation of variably functionalized cell walls containing protective pigments such as melanin, additional polymers such as xylomannan derivatives, and hydrophobic proteins such as hydrophobins.^[^ ^10^ ^]^

Mycelia adhere to two main growth strategies while colonizing the surrounding environment, based on their physiology and the availability of nutrients. They either undertake a strategy called *guerrilla* growth by developing explorative hyphae which are sparse, far‐reaching, fast‐growing and have a short lifespan, or undertake a strategy called *phalanx* growth by developing dense hyphal networks that slowly colonize the substrate.^[^ ^25^ ^]^ Hyphae are typically formed as singular cellular chains separated by septa, which act as walls between cells. Certain fungal species also exhibit the genomic ability to generate different types of hyphae that build spore‐bearing reproductive structures; sporocarps or mushrooms. In these cases, the thin‐walled generative hyphae, which carry spores and cytoplasm, are always present while the thick‐walled skeletal and binding hyphae, which provide structure and support to the fruiting body, may be lacking.^[^ ^26^ ^]^ These hyphae are capable of exquisite highly organized structures when forming mushrooms, which facilitate an extraordinary variety of shapes and expected differences in mechanical properties.^[^ ^27^ ^]^

Fungal colonies not only progress through predetermined stages, from spore germination to mature colony formation, but adapt to their surrounding environmental conditions by regulating their shape with a rich arsenal of biochemical mechanisms. For instance, fungal nutrient transport operates utilizing hyphal cytoplasmic flows^[^ ^24^ ^]^ and fungi may block specific hyphal pathways by closing septa to limit harm arising from damage to their structure or to direct resources to specific locations within the mycelium network.^[^ ^28^ ^]^ Variations in fungal morphology and physiology, associated with the vast biodiversity of this kingdom, allow for the selection of fungal species with desired traits, to generate living structures that may adapt to changes and thrive over time.

**Figure 1:**
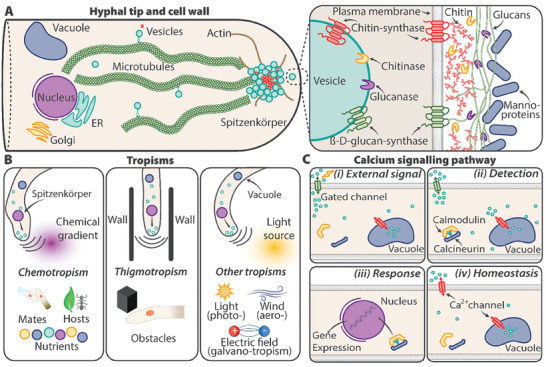
Directional hyphal growth and sensing. A) Schematic illustrates the manufacturing of a new cell wall. Secretory vesicles, originating from organelles such as the Golgi apparatus or the endoplasmic reticulum, travel to the hyphal apex via motor proteins on microtubules. Vesicles reach a subapical region and aggregate into the Spitzenkörper. Vesicles transport new plasma membrane to the growing tip in addition to transmembrane enzymes, such as chitin synthase, glucan synthases, chitinase, and glucanase, which in turn remodel the cell wall. Cell wall schemes depicting a typical hyphal cell wall for *Aspergillus fumigatus* are included in the inset. B) Various mechanisms that direct hyphal growth are shown: chemotropism, thigmotropism, and other tropisms. C) Ca^2+^ signaling pathways of fungi. Fungi sense their environment and rapidly respond to extracellular cues by activation of calcium signaling pathways. (i) Upon detection of an external stimulus, storage organelles in the cytosol release calcium, (ii) which is detected by Calmodulin and Calcineurin, (iii) forming a complex that influences gene expression. (iv) When no signal is present, intracellular Ca^2+^ homeostasis is maintained by ion transport across the plasma membrane and storage in organelles using channels, exchangers, and pumps.

One key aspect of regenerative living fungal structures depends on the organism's ability to direct hyphal growth. Hyphae grow via an intracellular biochemical process which initiates cell wall and membrane expansion at the hyphal tip,^[^ ^24^ ^]^ similar to the growth of pollen tubes^[^ ^29^ ^]^ and neurons.^[^ ^30^ ^]^ New hyphae extend from germinating propagules or branch from old hyphae through polarized intracellular mechanisms, which precisely direct hyphal formation and elongation.^[^ ^31^ ^]^ Localized traffic occurs at the hyphal tip, marked by the generation of proteins such as Spa2 and Bud6^[^ ^32^ ^]^ or GTPase Cdc42.^[^ ^33^ ^]^ Septins are then recruited and form rings, which interact with the cytoskeleton to connect the hyphal apex with organelles in distal cell regions and guide the dynamic organization of microtubules.^[^ ^34^ ^]^ The organized cytoskeleton supports the transport of secretory vesicles towards the apex to develop the Spitzenkörper, shown schematically in **Figure** **1**A.

The Spitzenkörper actively regulates directional hyphal growth by guiding the path of secretory vesicles from microtubules that then fuse with the plasma membrane at the apex^[^ ^35^, ^36^, ^37^, ^38^, ^39^ ^]^ and provide new material for tip expansion and elongation. They also transport glycoproteins and enzymes necessary for cell wall synthesis and remodeling, which are secreted extracellularly and contribute to apical growth by expanding the cell wall.^[^ ^40^ ^]^ This secretory pathway provides transmembrane enzymes, such as chitin‐ or glucan‐synthase, which are devoted to exporting monomers of chitin (N‐acetyl‐D‐glucosamine) and glucan (ß‐D‐glucose), to synthesize extending chains on the outer side of growing hyphae (Figure 1A). These polymers remodel and expand the cell wall as they move passively towards subapical regions where the matrix crosslinks.^[^ ^41^ ^]^ New hyphal growth relies on an elevated intracellular Ca^2+^ concentration at the hyphal tip, which is regulated by various calcium signaling pathways in fungi.^[^ ^42^ ^]^ The Ca^2+^ ions influence the fusion of the secretory vesicles with the plasma membrane by modulating the activity of SNARE receptors^[^ ^24^ ^]^ and therefore play a role in the directionality of tip growth.^[^ ^40^, ^43^ ^]^

Fungi sense their surrounding environment by detecting external signals, such as nutrient gradients, chemicals secreted by potential symbiotic partners, and environmental cues such as light and temperature changes. They direct hyphal growth through biochemical regulation and extension of hyphae to explore new directions or grow towards targets, as illustrated in Figure 1B. Various mechanisms influence the growth of mycelia. For example, chemotropism arises from environmental chemical gradients that fungi use to locate nutrients,^[^ ^44^, ^45^ ^]^ pheromones,^[^ ^46^, ^47^ ^]^ and potential hosts.^[^ ^48^, ^49^ ^]^ *Fusarium oxysporum* senses heme‐containing enzymes secreted by the roots of tomato plants, a potential host, via transmembrane proteins inducing mitogen‐activated protein kinase (MAPK) cascades and calcium signals to direct hyphal growth towards the host.^[^ ^49^ ^]^ When fungi grow along surfaces, they tend to move around obstacles in response to touch using thigmotropism. *Neurospora crassa*, for instance, can navigate around walls and corners thanks to a directional memory that is possible through the activity of the Spitzenkörper, turgor pressure,^[^ ^36^ ^]^ and potential cell wall mechanosensors.^[^ ^50^ ^]^ These mechanosensors may help regulate cell wall mechanical properties, such as stiffness and thickness, in response to physical stimuli. This mechanosensing mechanism, paired with chemical sensing through the accumulation of signaling molecules such as calcium ions or reactive oxygen species, drives hyphal penetration and entry of a host by inducing changes in the cytoskeleton and vesicle distribution.^[^ ^51^ ^]^ Other tropisms may arise from a variety of stimuli. Some examples include aerotropism, where fungi grow as a result of wind or gaseous oxygen gradients,^[^ ^52^ ^]^ phototropism, i.e., light‐directed growth,^[^ ^53^ ^]^ and galvanotropism, where fungi respond to an external electric field, growing towards anodes or cathodes depending on Ca^2+^ concentration and pH.^[^ ^54^, ^55^, ^56^ ^]^ In most cases, sensing mechanisms at the molecular level are still poorly understood, however, the signaling pathways are gradually being explored.

## 3 Fungal Signaling

Fungi use gradients in cellular ion concentration to generate signals in response to external stimuli, such as changes in light, availability of nutrients, presence of toxins, and variations in temperature or pH. They also respond to developmental cues such as signals for initiating spore formation, mating processes, or transitioning between growth phases like vegetative growth to fruiting body formation. Calcium and protons are some of the most frequently used ions with fungal signaling functions, initialized through changes in these ion concentrations, that then direct fungal growth and redirect resources. In fungi, cytoplasmatic calcium levels are maintained approximately ten thousand times lower than extracellular levels which can reach 10^−3^ m, due to the activity of transmembrane proteins that form Ca^2+^‐permeable channels, Ca^2+^‐pumps, and Ca^2+^‐exchangers.^[^ ^57^ ^]^ These proteins are located on the hyphal cell and organelle membranes and both actively and passively transport calcium ions to maintain cytoplasmic homeostasis. Active hyphal growth depends on a tip‐localized calcium concentration, likely due to the role of calcium ions in vesicle fusion and enzyme activation via the signaling pathway.^[^ ^58^ ^]^ The calcium signaling pathway for fungi is outlined in Figure 1C. Excess calcium ions are expelled from the hyphae or stored in the endoplasmic reticulum, Golgi apparatus, or vacuole organelles, resulting in a reserve of ions that can, when needed, rapidly perturb calcium homeostasis.^[^ ^57^, ^59^ ^]^

Detection of external signals by hyphal transmembrane proteins triggers a signaling cascade resulting in the release of calcium from these organelles and a rapid increase in cytoplasmic calcium concentration. This increased intracellular calcium concentration is detected by calcium‐sensitive proteins, such as calmodulin and calcineurin which interact to form an active phosphatase in the cytoplasm. This new phosphatase removes phosphate groups from cytoplasmic nuclear factors to activate transcription factors and in turn regulate gene expression in a process called dephosphorylation. The ability of this phosphatase to control cytoplasmic calcium and sense changes in its concentration facilitates rapid regulation of gene expression to orientate hyphae,^[^ ^60^, ^61^ ^]^ respond to stress,^[^ ^62^, ^63^ ^]^ or adapt to environmental changes.^[^ ^64^ ^]^

Proton concentration across hyphal membranes is also critical to fungi signaling, transmitting information through the mycelium by leveraging generated electrical activity.^[^ ^56^, ^65^, ^66^, ^67^ ^]^ Trans‐hyphal current is associated with hyphal polarization and branching,^[^ ^56^ ^]^ identification and regulation of nutrients, and intracellular pH.^[^ ^68^ ^]^ Molecular mechanisms facilitating such regulation are not well characterized, however, fungi are known to express pump‐ and channel‐protein complexes for active and passive proton transport to regulate proton concentration within the cytosol.^[^ ^69^, ^70^, ^71^ ^]^ Hyphal tips act as proton sinks, promoting inward apical proton flux and, in doing so, generating an electric field that propagates several hundred microns back into the hyphae, before reaching an area of depolarization where protons are secreted.^[^ ^72^ ^]^ This effect can be attributed to uneven distribution of proton transport systems along the hyphae.^[^ ^55^, ^72^, ^73^, ^74^, ^75^ ^]^ It is due to these transport systems that hyphae can propagate current over long distances, transmitting the electric signal to adjacent cells. This effect continuously disrupts transmembrane potential, in a fashion similar to the pulse‐like action potential of neurons.^[^ ^67^, ^76^, ^77^ ^]^ Such action potential‐like behavior likely arises from intracellular ion waves from calcium and proton signaling pathways initiated by external stimuli,^[^ ^77^ ^]^ which may drive the propagation of complex signals through large mycelial structures.^[^ ^66^ ^]^ Regulation of environmental conditions, such as ion availability, is consequently integral to harnessing the full potential of fungal living materials, predicated on a thorough understanding of fungal electrophysiological signals.

## 4 Recording Fungal Signals

From fluorescent probes and indicators to intra‐ and extra‐cellular recording probes, various methods have been instrumental in exploring ion dynamics in fungal cells. Visualization of ion concentrations and flows is readily achieved through optical imaging of fungal species. Dyes containing single excitation fluorophores, such as Fluo‐3, Fluo‐4, and their membrane permeable acetoxymethyl (AM) ester variants, are effective for intracellular imaging of Ca^2+^ ion concentrations^[^ ^78^ ^]^ even in response to external stimuli such as plasma treatment.^[^ ^79^ ^]^ Multiple excitation (ratiometric) probes like Indo‐1 or cSNARF‐1, with distinct profiles for ion‐sensitive and insensitive wavelengths, offer greater versatility in the study of micro‐scale details, such as Ca^2+^ gradients in hyphal tips^[^ ^80^ ^]^ or recording of cytoplasmic pH.^[^ ^81^, ^82^ ^]^ However, dye‐based indicators struggle to penetrate cell membranes, sequestering within organelles and leading to gradient‐like artefacts and shortened time scales, or photobleaching.^[^ ^83^, ^84^ ^]^

Recently, genetically encoded calcium indicators (GECI) have emerged as dye replacements. Three main indicators, namely R‐GECO, GCaMP, and aequorin‐based probes, offer distinct advantages over chemical dyes; fungal cells generate calcium indicators in situ allowing for higher spatial and temporal resolution.^[^ ^85^ ^]^ R‐GECO, based on a red fluorescent protein (RFP) characterized by a red‐shifted emission, enables deep‐tissue imaging and has been employed to investigate wavy propagation and blinking calcium signals in externally stimulated *Aspergillus nidulans*.^[^ ^86^ ^]^ GCaMP probes, typically sourced from the fusion of calmodulin, M13 peptide, and green fluorescent protein (GFP), emit green fluorescence upon binding to calcium ions.^[^ ^87^ ^]^ Live‐cell imaging of fungal cells shows Ca^2+^‐specific responses in *Candida albicans* exposed to membrane, osmotic, and oxidative stressors^[^ ^88^ ^]^ and *Saccharomyces cerevisiae* in response to pheromones.^[^ ^89^ ^]^ Aequorin, a bioluminescent protein that emits blue light upon binding with calcium, reveals an immediate influx of extracellular Ca^2+^ in *C. albicans* when exposed to alkaline stress^[^ ^90^ ^]^ and in *Aspergillus fumigatus* when exposed to high concentrations of the antifungal drug caspofungin.^[^ ^63^ ^]^ While both chemical dyes and GECIs offer valuable insights into ionic dynamics, both have limitations. GECIs require species‐specific genetic manipulation and are still liable to some problems associated with chemical dyes, such as specificity and photobleaching. Achieving a comprehensive understanding of fungal cellular signaling consequently requires a variety of investigation techniques.

## 5 Bioelectrical Activity in Fungi

**Figure 2:**
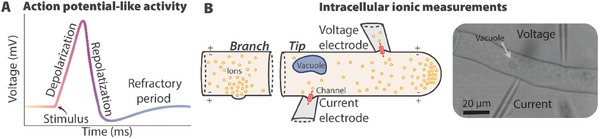
Bioelectrical signaling in fungi. A) Action potential‐like representation of a typical intracellularly measured voltage spike. B) Micropipette probe for intracellular ion flux measurements. (i) A scheme of the proton concentration and flow throughout a hypha and subsequent intracellular voltage measurement. (ii) Image of a microelectrode in a *Neurospora crassa* hypha to measure voltage and inject current. Image reproduced with permission.^[^ ^108^ ^]^ Copyright 2007, R. Lew.

Fungal electrophysiological and action potential‐like activity is currently recorded using techniques including intracellular and extracellular measurements^[^ ^66^ ^]^ (**Table** **1**). Action potential‐like activity represented as spontaneous voltage shifts includes three key phases: depolarization, repolarization, and a refractory phase,^[^ ^102^ ^]^ all observed at multiple length scales within fungal microstructures (**Figure** **2**A).

**Table 1:** Techniques for recording electrical signals in fungi and plants.

Technique	Method	Description	Advantage	Limitation	Electrode Type	Species
Intracellular measurement	Patch‐clamp	Used in electrophysiology to study the electrical properties of individual cells by forming a seal between a glass pipette and cell membrane to control the ion channels.	High sensitivity. Precise measurement.	Time‐consuming. Requires specialized equipment and skilled operator. Allows the study of a limited number of cells simultaneously. Safe for short‐term measurements. Invasive.	Glass micropipette	*Phycomyces blakesleeanus* ^[^ ^91^, ^92^ ^]^ *Aspergillus niger* ^[^ ^93^ ^]^ *Arabidopsis thaliana* ^[^ ^94^, ^95^ ^]^ *Pleurotus ostreatus* ^[^ ^67^ ^]^ *Armillaria bulbosa* ^[^ ^67^ ^]^ *Neurospora crassa* ^[^ ^77^ ^]^
Extracellular measurement	Needle electrodes	Used to detect electrical signals within biological tissue by inserting fine metal needle electrodes into specific regions.	Easy to use. Versatile.	Allows the study of a limited area of the biological tissue simultaneously. Invasive.	Pt/Ir	*Ganoderma lucidum* ^[^ ^96^ ^]^
					Pt	*Laccaria bicolor* ^[^ ^97^ ^]^
					Ir‐coated stainless steel	*Pleurotus ostreatus* ^[^ ^98^ ^]^ *Omphalotus nidiformis* ^[^ ^99^ ^]^ *Flammulina velutipes* ^[^ ^99^ ^]^ *Schizophyllum commune* ^[^ ^99^, ^100^ ^]^ *Cordyceps militaris* ^[^ ^99^ ^]^ *Ganoderma resinaceum* ^[^ ^101^ ^]^ *Pleurotus djamor* ^[^ ^102^, ^103^ ^]^
					Stainless‐steel	*Chlorophytum comosum* ^[^ ^104^ ^]^
	Vibrating Probe	Used to detect the physical or chemical behavior of a sample by mechanically oscillating the probe at its resonant frequency.	High sensitivity. Versatile. Non‐invasive.	Requires specialized equipment and skilled operator. Sensitive to environmental factors. Not suitable for deep tissue measurements.	Glass micropipette and Pt‐black	*Achlya bisexualis* ^[^ ^55^, ^105^ ^]^ *Neurospora crassa* ^[^ ^55^, ^105^ ^]^ *Basidiobolus ranarum* ^[^ ^105^ ^]^ *Allomyces macrogynus* ^[^ ^105^ ^]^ *Mucor mucedo* ^[^ ^55^ ^]^ *Schizophyllum commune* ^[^ ^55^ ^]^ *Aspergillus nidulans* ^[^ ^55^ ^]^ *Gigaspora margarita* ^[^ ^106^ ^]^ *Trifolium repens* ^[^ ^106^ ^]^ *Daucus carota* ^[^ ^106^ ^]^
	Surface electrodes	Used to detect electrical activity directly from the surface of the biological tissue.	Easy to use. Non‐invasive. Safe for long‐term measurements.	Sensitive to environmental factors. Limited spatial resolution.	Stainless‐steel grids	*Chlorophytum comosum* ^[^ ^104^ ^]^
					Graphite plates	*Vigna radiata* ^[^ ^107^ ^]^ *Cicer arietinum* ^[^ ^107^ ^]^

Intracellular recording techniques utilizing glass microelectrodes inserted into hyphae can be used to measure ion gradient‐induced electric fields in *Neurospora crassa*.^[^ ^77^ ^]^ Such measurements indicate a resting membrane potential (originating from ion movement across ion channels and transporters in the cell membrane^[^ ^109^ ^]^) of approximately −190 mV, indicating that the concentration of ions outside the cell is higher than that inside. This was followed by a slow depolarization over 100 s to approximately −90 mV. After reaching the threshold, the voltage peaked at around −30 mV.^[^ ^77^ ^]^ The magnitude of these spontaneous oscillations or action potential‐like activities was approximately −40 mV for 1–2 min across various experiments.^[^ ^77^ ^]^ This intracellular recording technique is accurate but time‐consuming, labor intensive, and only possible on short time scales.

Many researchers prefer to observe extracellular signals in mycelia^[^ ^67^ ^]^ or fruiting bodies,^[^ ^66^ ^]^ for which the signal is distributed over a larger biological structure. This technique is more straightforward and enables longer recordings. Voltage in mycelia ranges from approximately 5−50 mV in *Arethusa bulbosa and Pleurotus ostreatus*, with a frequency of 0.5−5 Hz.^[^ ^67^ ^]^ At this larger (fruiting body) scale, two types of action potential‐like impulses were identified: high frequency (period of 2.6 min) with an average amplitude of 0.88 mV and low frequency (period of 14 min) with an average amplitude of 1.3 mV.^[^ ^66^ ^]^ Robust experimental setups at the fruiting body scale are challenging and issues with accuracy persist, as described by Blatt et al.^[^ ^110^, ^111^ ^]^ Recommended improvements to experimental methodologies include recognizing the biological origins of voltage transmission and designing experiments around these. Electrode types should also be considered and precautions including noise control or adoption of better techniques, such as intracellular microelectrodes, taken due to the potential for interference from both biological and non‐biological sources. Substrates, such as aqueous gels and agar, can impact ionic mobility and generate Donnan potentials. Compatibility between electrolyte solutions and cellular components is also essential to avoid unintended cellular damage. Incorporating intercellular measurements could facilitate a better understanding of cellular processes during experiments. Adopting these considerations in experimental protocols would enhance the reliability and reproducibility of electrophysiological studies, facilitating deeper insights into biological electrical dynamics.

While measuring bioelectrical activity, researchers have also noticed changes in fungal electrical responses to different environmental stimuli such as humidity,^[^ ^97^ ^]^ temperature,^[^ ^97^ ^]^ light,^[^ ^112^, ^113^ ^]^ nutrients and chemicals,^[^ ^66^, ^113^, ^114^ ^]^ and mechanical loading.^[^ ^101^, ^114^ ^]^ However, it must be noted that, although serving as valuable proof‐of‐concepts, these results do not represent interpretable data or exhibit repeatability. As we look to the future, understanding the implications of these voltage spikes will help us understand fungal physiological changes outside the experimental environment.

## 6 Spike Sorting

Biochemical processes influence voltage spikes, and noise typically arises from averaging the active fungal cells within tens to hundreds of extracellularly signaling hyphae. Signals often undergo deconvolution using spike sorting to identify impulses and their origin.^[^ ^115^, ^116^ ^]^ Background noise is first removed using techniques such as high‐pass filtering for low‐frequency components representing background noise or slow drifts in the signal.^[^ ^117^ ^]^ This step isolates action potentials generated by individual hyphae.^[^ ^118^, ^119^ ^]^ Spike detection techniques, including thresholding to capture only higher voltage spikes, are then employed. Each individual event is identified and marked by calculating quantitative parameters for each spike, after which spikes with similar features are grouped into clusters.^[^ ^120^ ^]^ However, traditional signal processing methods face challenges in accurately classifying these signals due to the lack of clear signal baselines and the complexity of the data. Manual sorting has been shown to have high variability in spike labels and error rates in excess of 23%, which only pales in comparison to the increasing volume of data arising from large electrode arrays.^[^ ^121^, ^122^ ^]^ Incorporating machine learning and deep learning offers distinct advantages in this context, as these techniques excel at handling large datasets, identifying complex patterns, and automating and ensuring consistency in data processing.

Cluster analysis methods in machine learning facilitate the grouping of spikes with similar features, making data interpretation more manageable. For instance, k‐means clustering assigns each spike to the nearest cluster centroid, organizing spikes into distinct groups based on their features.^[^ ^123^ ^]^ This method is a powerful tool for classifying intracellular images of Ca^2+^ oscillation patterns in rat neurons, revealing diverse response signals.^[^ ^124^ ^]^ Nearest‐neighbor methods sort data points based on the most common classes of their nearest neighbors^[^ ^125^ ^]^ and have effectively decoded delicate wound‐induced electrical signals in plants, achieving high classification accuracy.^[^ ^126^ ^]^ Bayesian optimization is flexible, providing the maximum and minimum of an objective function with few evaluations.^[^ ^117^ ^]^ This method has been essential for dealing with the variability in plant electrophysiology when the plant is electrically stimulated,^[^ ^127^ ^]^ and in the more nuanced situations such as plant‐pathogen interactions when a host plant faces a fungal infection.^[^ ^128^ ^]^ Sparse coding represents data as a linear combination of basic functions, enforcing sparsity in coefficients and useful for feature extraction but potentially struggling with noisy or incomplete datasets.^[^ ^129^ ^]^ However, these methods face limitations such as sensitivity to initial cluster centers, handling datasets with many variables or features, intensive computations, and noisy datasets, depending on the chosen clustering method. Nonetheless, their use in categorizing neural action potentials and the less easily identified “plant action potentials” holds strong promise for comprehending fungal electrophysiological signals, an area still in its early stages of research.

Processing fungal signals with deep learning algorithms and spike sorting techniques represents a unique approach to the development of mycelium‐based, sustainable, smart materials. Deep learning may be able to decode and analyze extracellular activity in mycelial tissues^[^ ^116^ ^]^ and classify spikes based on their corresponding individual cells, similar to how neural spikes are detected and recorded in brain sensors.^[^ ^130^ ^]^ Information can be captured from individual cells using micro‐electrode arrays which already enable the capture of spike activity from one or multiple neurons through hundreds of electrodes.^[^ ^131^, ^132^ ^]^ Alongside micro‐electrode arrays, 3D printing techniques, such as two‐photon lithography, have generated micro‐hook arrays for plants allowing sensing of delicate leaf environments.^[^ ^133^ ^]^ Large datasets generated from these types of arrays would be best processed via fully automated spike‐sorting algorithms utilizing both supervised and unsupervised approaches which have already been developed for brain signals. For instance, the SpikeDeep‐Classifier efficiently isolates and extracts individual neuron activity amidst background noise, achieving high accuracy, helping analyze human brain behavior and network properties.^[^ ^130^ ^]^ Advanced deep spike sorting algorithms and deep learning techniques may hold the key in interpreting and understanding the bioelectrical activity of mycelium‐based composites in response to environmental stimuli, such as nutrient deprivation or physical pressure (e.g., applying load to the mycelium network). This could revolutionize engineered living materials and drive further development.

## 7 Harnessing Signaling in Functional Living Materials

**Figure 3:**
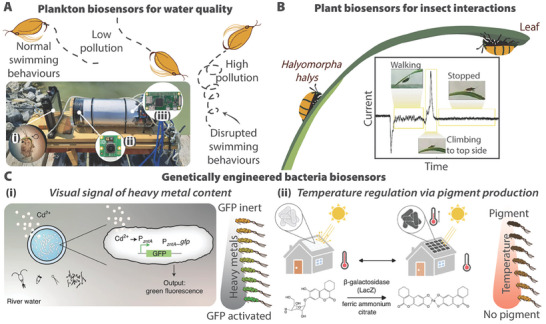
Some recent examples of biosensors. A) Schematic of *Daphnia* plankton as biosensors. Motion tracking of plankton indicates pollution concentrations via disrupted swimming behaviors, such as spinning or lurching.^[^ ^135^, ^140^, ^141^ ^]^ Inset shows an experimental set‐up to monitor (i) *Daphnia* swimming through a chamber using a (ii) camera and (iii) Raspberry Pi controller. Image modified under terms of the CC‐BY license.^[^ ^135^ ^]^ Copyright 2023, W. Rajewicz et al., published by Springer Nature. B) Bioelectric signals measured as an insect walks on a leaf. Inset depicts recorded currents over time from a bug walking, climbing, and stopping on a leaf. Inset reproduced with permission.^[^ ^137^ ^]^ Copyright 2023, S. Armiento et al., under exclusive license to Springer Nature America, Inc. C) Genetically engineered *Escherichia coli* for biosensing and self‐regulation. (i) Engineered *E. coli* for visual signaling of heavy metal content, such as cadmium,^[^ ^138^ ^]^ via activation of a green fluorescent protein (GFP). Inset reproduced with permission.^[^ ^138^ ^]^ Copyright 2021, T. Tang et al., under exclusive license to Springer Nature America, Inc. (ii) Engineered *E. coli* for temperature regulation through the production of a light‐absorbing pigment at colder temperatures.^[^ ^139^ ^]^ Inset reproduced under terms of the CC‐BY license.^[^ ^139^ ^]^ Copyright 2023, L.L. Xiong et al., published by Wiley‐VCH.

Two things are needed to harness the signals of living organisms for human benefit: 1) a living organism must react to changing environmental conditions in a measurable way, and 2) a means of interpreting the biosensor's signal must exist. Signal types may range from simple binary indicators, like a dog barking upon detecting illicit drugs, to complex electrical signals caused by intracellular ion flows. Simple biological sensors date back centuries to human use of sentinel animal species to detect ecological danger to humans. For example, canaries react to high carbon monoxide levels more quickly than humans and were consequently used by British coal miners as early indicators of dangerous toxic gas levels.^[^ ^134^ ^]^ From simple examples like this one, the principle of using organism signaling to monitor changing environmental conditions is now receiving renewed interest among the scientific community for increasingly complex tasks. Recent examples of harnessing signaling in living organisms involve more and more complex signals and measurement methods. For example, a recent developmental concept involves monitoring the changes in the movement of *Daphnia* plankton to monitor water salinity levels, as shown in **Figure** **3**A.^[^ ^135^ ^]^ Mussel shell assemblies can also be used to monitor water quality; their gills act as a water filter, removing particulates and pollution when the shell opens. Insights into changes in water quality can be generated by monitoring the gape angle of these mussel shells over time with hall sensors.^[^ ^136^ ^]^ Plant‐insect interactions can be monitored through the triboelectric charge effect of moving insects, which results in a changing current on the plant leaf (Figure 3B).^[^ ^137^ ^]^ In bacteria, there are many possibilities for biosensing due to the opportunities provided by genetic engineering. Some examples are the detection of metal ions like cadmium^[^ ^138^ ^]^ in water or the measurement of ambient temperature^[^ ^139^ ^]^ (Figure 3C).

Fungi also hold great promise as biosensors; work in this field is in its infancy, however, applications with considerable potential have already been demonstrated. *Laccaria bicolor* mushrooms exhibit an electrical potential exceeding 100 mV in response to rainfall events in forest settings, exhibiting great promise for ecological monitoring.^[^ ^97^ ^]^ Such measurements were conducted using subdermal needle electrodes inserted into mushroom caps and are distinct from measured negligible electric potentials for non‐living reference mushrooms. Other researchers seek to electrically interface with underground mycelium networks using graphite electrodes embedded at strategic access points in forests.^[^ ^142^ ^]^ Spontaneous electric activity was measured as electric potential and impedance across a “mycelium bridge” connecting two agar‐based monitoring nodes separated by a gap. In addition to monitoring, signal sorting and analysis could facilitate the creation of feedback loops to optimize growth. For example, the “Speaking Mushroom Approach” has been proposed as a biosensor solution to maintain optimal lighting conditions for mushroom formation.^[^ ^112^ ^]^ In this case, the grow light intensity is regulated by a control system programmed to interpret changes in measured fungal bioelectric signals.^[^ ^112^ ^]^ The monitoring of mycelium‐ and mushroom‐based electrical activity, if further developed, could result in biosensing applications related to changes in humidity, temperature, and potentially many other conditions yet to be investigated.

**Figure 4:**
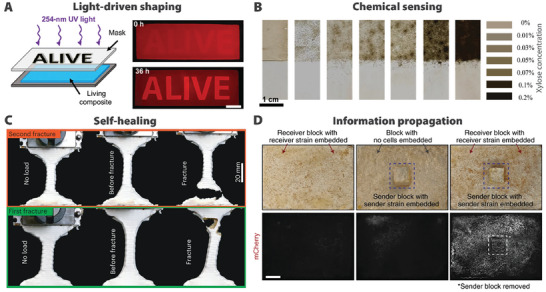
Examples of biosensor materials made using fungi. A) Illustration of the mechanism by which *Saccharomyces cerevisiae* cells proliferate inside a hydrogel based on exposure to UV light. The cells do not proliferate in the area covered by the mask, leading to swelling variations that provide contrast to the letters of the word “ALIVE.” Reproduced under terms of the CC‐BY license.^[^ ^144^ ^]^ Copyright 2020, L.K. Rivera‐Tarazona et al., American Association for the Advancement of Science (AAAS). B) Color changes in *Aspergillus niger* films in response to increasing xylene concentrations.^[^ ^145^ ^]^ Reproduced with permission.^[^ ^145^ ^]^ Copyright 2023, Elsevier. C) Mycelium‐laden 3D‐printed hydrogel demonstrates robust self‐healing, evident by the shifting fracture line from the first test to the retest after healing. Reproduced with permission.^[^ ^16^ ^]^ Copyright 2022, Gantenbein et al., under exclusive license to Springer Nature Limited. D) Mycelium composite loaded with engineered bacteria demonstrates the ability to receive signals from a transmitter block. Reproduced with permission.^[^ ^17^ ^]^ Copyright 2022, R. M. McBee et al., under exclusive license to Springer Nature Limited.

Thanks to robust environmental sensing capabilities, fungi have been considered for various applications in living materials and structures. Modified *S. cerevisiae* yeast cells embedded into polyacrylamide hydrogels demonstrate proliferation‐induced shape change in response to UV (**Figure** **4**A) and blue light.^[^ ^144^ ^]^ Genetically engineered *Aspergillus niger* mycelia grown in liquid culture can be vacuum‐filtered into living membrane materials, which change color on exposure to xylose in wastewater (Figure 4B).^[^ ^145^ ^]^ Research interest in the integration of living functionalities into structures, such as films, hydrogels, and composite materials, implementing filamentous fungi has recently exploded. Pure mycelium films fabricated as leather substitutes can survive dry conditions and self‐heal damage within two days.^[^ ^22^ ^]^ *Ganoderma lucidum* mycelium in 3D‐printing hydrogel ink similarly shows profound self‐healing characteristics (Figure 4C).^[^ ^16^ ^]^ Mycelium‐wood composite blocks hybridized with engineered *Pantoea agglomerans* bacteria can produce mCherry fluorescent reporters when in contact with composite blocks containing quorum sensing molecule Acyl Homoserine Lactone AHL (Figure 4D).^[^ ^17^ ^]^ This pioneering work hints at the possibility of maintaining living fungi within structures to facilitate biosensing functionalities.

## 8 Future Work in Fungal Signaling

Fungal sensors may exhibit considerable advantages over traditional electronic sensors, namely low manufacturing energy and cost, broad sensing capabilities in a single unit, and well‐demonstrated self‐assembly and self‐repair capabilities.^[^ ^14^, ^16^, ^146^ ^]^ To fully tap into the potential of fungi as biosensors, the following research areas need to be addressed: 1) understanding and interpretation of fungal signals, 2) creation of embedded fungal sensors, and 3) incorporation of living fungi in structures. A well‐developed understanding of these research areas will enable the use of embedded fungal cells as living biosensors in materials, for use in structural monitoring applications.

Research on electrical signal measurement and interpretation in fungi is still in its early stages. Measurement of spontaneous extracellular potential at the local level has already been demonstrated,^[^ ^66^, ^100^ ^]^ but systematic identification of the signaling phenomena is still missing.^[^ ^111^ ^]^ Measured signals also show high variability, complicating signal interpretation and data extraction.^[^ ^66^, ^67^ ^]^ The spontaneous signal baseline must be systematically characterized and understood before signal changes in response to environmental stimuli can be accurately detected. Characterizing the signal baseline requires carefully controlled experiments that do not contain electrical noise from electrodes or conductive media vibrations.^[^ ^111^ ^]^ Only once the signal baseline has been comprehensively characterized, will the matching of input stimuli to signal response be possible. Experiments to date, demonstrating action potential responses to input stimuli, such as application of load, light, and chemicals^[^ ^101^, ^147^ ^]^ are important starts, but much more data must be collected in this area. Fungal signal responses to stimuli must also be carefully controlled, to eliminate possible confounding electric phenomena such as the triboelectric effect. Large datasets will help interpret electric signals through sorting and machine learning, enabling stimuli‐response relationships, generating predictive power for signal interpretation.

Once a thorough understanding of fungal electrical signals has been developed, embedded sensors must be created. Such research is currently lacking in its entirety. Current measurement of electric potential relies on invasive electrode systems that could potentially damage fungal organisms and compromise signal interpretation.^[^ ^66^, ^113^ ^]^ Further, since electrodes are manually placed into contact with the organism, the approach lacks precision, both in distance between electrodes, and depth of insertion. Scalability represents an even greater challenge; measurement of potential difference at contact points and the attainment of a representative signal requires multiple electrode pairs, with the management of increasing numbers of electrodes quickly becoming challenging. New organism interfacing approaches must be explored for more complex applications, such as in situ monitoring of structures. Using 3D printing techniques to embed electrodes within mycelial networks and fabricate bio‐compatible electrodes could represent one solution to this problem. For example, mycelium‐laden ink co‐extruded with electrically conductive ink could yield multi‐material systems able to be interfaced at any point in the network. This represents a non‐invasive solution that could enable precise measurement of mycelium network signaling at contact points with the material without inserting electrodes or damaging the organism. New scalable approaches could also explore methods to transmit signals from embedded electrodes to measuring devices wirelessly.

With fungal sensors, there is a challenging question of how they could be integrated into large‐scale structures while being kept alive. Academic and commercial interest is currently focused on the use of mycelium to bind lignocellulosic material into composites^[^ ^148^, ^149^, ^150^, ^151^ ^]^ and films,^[^ ^152^, ^153^, ^154^ ^]^ which are then deactivated. The use of living fungi in materials presents unique challenges beyond those typically associated with fungal materials. The fungus must be kept alive within a structure and its essential bio‐chemical pathways supported if its signaling is to be harnessed. This means supplying the organism with nutrients, water, and oxygen for the entire service life of the material. Such support is currently achieved by incubating the host material under wet conditions in nutrient‐rich media, where self‐repairing or sensing functionalities are desired.^[^ ^14^, ^16^, ^22^ ^]^ An alive and hydrated mycelium exhibits low wet strength, as does its substrate. It may also emit odors and Volatile Organic Compounds (VOCs)^[^ ^155^ ^]^ and attract insects.^[^ ^156^ ^]^ Perhaps an even bigger challenge is that mycelium digests the lignocellulosic substrates that it grows on, likely resulting in decreased material strength over time. Species‐specific solutions tailored to the biochemical needs of a given mycelium are necessary to integrate living fungal networks into structures. These may consist of multi‐material additive manufacturing techniques, which tailor the structure and material to fit the needs of the living sensor.

Living structures hold a discrete advantage over man‐made structures, in their ability to sense their surroundings and react to the ever‐changing environment. This adaptability makes living structures resilient and resistant to changing conditions, enabling them to, in some cases, achieve incredible sizes and lifespans. The giant *Armillaria ostoyae* fungus occupies approximately 10 km^2^ of soil in Oregon, U.S.A. and could be up to 8650 years old,^[^ ^8^, ^157^ ^]^ over three times older than the biggest single‐stem living tree, the General Sherman Sequoia tree from California, which is 84 m tall and 12 m wide.^[^ ^158^ ^]^ Humans have attempted to replicate this longevity in structures through the use of stronger materials, and structural health monitoring systems, but still cannot match the resilience and adaptability of robust living organisms. A lack of adaptability means that structures must be over‐engineered to operate safely within an unpredictable range of loads and environmental conditions. How could we progress towards manufacturing adaptive living materials? As thoroughly discussed in this paper, fungi show remarkable potential for integration in materials, to endow them with biosensing capabilities. We consequently propose combination of the shape freedom of additive manufacturing with the signaling activity of mycelium to “AM‐IMATE” built structures.

## Conflict of Interest

The authors declare no conflict of interest.
